# Variability of total step activity in children with cerebral palsy: influence of definition of a day on participant retention within the study

**DOI:** 10.1186/s13104-016-2218-9

**Published:** 2016-08-20

**Authors:** Nichola C. Wilson, Suzie Mudge, N. Susan Stott

**Affiliations:** 1 Department of Surgery, Faculty of Medical and Health Sciences, The University of Auckland, Auckland, New Zealand; 2 Paediatric Orthopaedic Department, Auckland City Hospital, Auckland, New Zealand; 3 Health and Rehabilitation Research Institute, Auckland University of Technology, Auckland, New Zealand

**Keywords:** Accelerometry, Physical activity, Physical disability, Youth

## Abstract

**Background:**

Activity monitoring is important to establish accurate daily physical activity levels in children with cerebral palsy (CP). However, few studies address issues around inclusion or exclusion of step count data; in particular, how a valid day should be defined and what impact different lengths of monitoring have on retention of participant data within a study. This study assessed how different ‘valid day’ definitions influenced inclusion of participant data in final analyses and the subsequent variability of the data.

**Results:**

Sixty-nine children with CP were fitted with a StepWatch™ Activity Monitor and instructed to wear it for a week. Data analysis used two broad definitions of a day, based on either number of steps in a 24 h monitoring period or the number of hours of recorded activity in a 24 h monitoring period. Eight children either did not use the monitor, or used it for only 1 day. The remaining 61 children provided 2 valid days of monitoring defined as >100 recorded steps per 24 h period and 55 (90 %) completed 2 valid days of monitoring with ≥10 h recorded activity per 24 h period. Performance variability in daily step count was lower across 2 days of monitoring when a valid day was defined as ≥10 h recorded activity per 24 h period (ICC = 0.765) and, higher when the definition >100 recorded steps per 24 h period (ICC = 0.62). Only 46 participants (75 %) completed 5 days of monitoring with >100 recorded steps per 24 h period and only 23 (38 %) achieved 5 days of monitoring with ≥10 h recorded activity per 24 h period. Datasets of participants who functioned at GMFCS level II were differentially excluded when the criteria for inclusion in final analysis was 5 valid days of ≥10 h recorded activity per 24 h period, leaving datasets available for only 8 of 32 participant datasets retained in the study.

**Conclusion:**

We conclude that changes in definition of a valid day have significant impacts on both inclusion of participant data in final analysis and measured variability of total step count.

## Background

Cerebral palsy (CP) is the commonest cause of physical disability in childhood, with a prevalence of 2.11/1000 live births [1]. Children with CP have impaired gross motor function which contributes to reduced activity levels compared to their typically developing peers [2–4]. The functional ability of children with CP can be classified by the Gross Motor Function Classification System (GMFCS), a valid and reliable 5 level system which classifies gross motor function of these children from I (least involved) to V (most severely involved) [5–7]. Ambulatory children with CP who function at GMFCS level I, II or III, have levels of walking activity which are between 20 and 60 % that of their typically developing peers, with an average daily step count of 8440 step (range 7478–9498) [2].

Physical activity in childhood is increasingly being recognised as important for children with CP to maintain optimum health throughout their lifespan [3, 8–11]. Therefore, there is increased interest in using activity monitors in these children to understand how different interventions in the lower limb might impact on intensity and amount of walking activity in the community. Accelerometers are the device of choice in the neurologic population because they are more reliable for step detection than pedometers and can capture a wider range of information, including duration of activity, step rate and intensity of activity [12]. The StepWatch™ Activity Monitor is one such device and is a sealed waterproof, microprocessor controlled device that uses a combination of acceleration, position and timing to detect steps. To date, the StepWatch™ Activity Monitor has been used to quantitate daily activity levels in children with CP and adults with neurological disorders, to assess activity related change after an intervention, [13] and as an outcome measure in small clinical trials [14].

The reported accuracy of step detection by the Step Watch™ Activity Monitor is 99 % when compared to ‘manual counting’ in both non-disabled adults [15] and children with CP [2]. This accuracy includes both indoor settings and controlled outdoor settings [15]. Further, the Step Watch™ Activity Monitor has been shown to be more accurate than other accelerometers in the detection of steps in the presence of a slow or shuffling gait or rollator use [16–18]. As such, the Step Watch™ Activity Monitor is regarded as one of the most accurate accelerometers in the neurologic population and has been used as a criterion standard against which other monitors are compared [19]. However, the majority of studies test the variability in measurement of step activity in a researcher controlled environment and in comparison with a gold standard. Any variation in step detection can then be attributed to the device, not the participant.

In the free-living natural environment, variability in step activity from day to day is a consequence not only of measurement error in the device but also the variation that occurs as an interaction of the person’s choices and behaviour and the environment. In addition, participants may inadvertently confound data collection by removing a monitor during specific activities or putting the monitor on incorrectly for periods of time, potentially changing the sensitivity of step detection. All of these factors combined lead to what has been termed ‘performance variability’ in the free living environment [20]. Researchers can generally not influence how and when the monitor is worn in the community but can influence the final dataset depending on the way they analyse the raw step activity data, which requires decisions about whether to include or exclude 24 h periods of monitoring when there appears to be very low step activity or reduced hours of activity [21].

Not all studies clearly report their decision making on the inclusion/exclusion of patient data from the final analysis and others adopt differing approaches to dealing with datasets when the monitor has recorded lengthy time periods of non-activity. For example, early studies using the Step Watch defined valid data collection as when the monitor had recorded at least 8 h of clearly defined step activity over a 24 h period [22] or when there was less than 3 h of ‘inadequate’ monitoring during the daytime hours of 6:00 a.m.–10:00 p.m. Inadequate monitoring was defined as wearing the monitor upside down, not wearing the monitor, or wearing the monitor incorrectly on the ankle (not in the correct plane) [2]. Other studies have defined a day of monitoring as 10 h of continuous recorded step activity during a 24 monitoring period [23–26]; however, one study included data in the analysis if more than 100 steps was recorded on the monitor during the 24 h monitoring period [27].

In children with CP, Ishikawa et al. have argued for extended periods of activity monitoring, with variation in length of monitoring based on GMFCS levels. The authors of that study defined an acceptable G coefficient as >0.8 (similar to an ICC of >0.8). Their reported minimum number of days to achieve a G coefficient of >0.8 for total daily step count for children aged 6–14 was six for GMFCS I, five for GMFCS II and four for GMFCS III [28]. However, such prolonged periods of monitoring have the potential to adversely affect subject compliance in a study, particularly in the disabled population.

We are interested in developing a study to look at activity monitoring as a primary end-point after surgical intervention in children with CP. The primary goal of this study was therefore to determine how different definitions of valid data collection over a 24 h period influenced the exclusion of participant data from study analysis and whether any bias would be introduced into the results by changing definitions of a valid day. A secondary goal was to determine the performance variability of measures of total step count over a 2 day period of monitoring in the free living environment, using the two common definitions of a valid day present in the literature.

## Methods

### Participants

The data for this study were collected as part of two studies, both approved by the Northern X Regional Ethics Committee and the ADHB Research Office and conducted over a period of 3.5 years. Inclusion criteria were children with CP, GMFCS levels I to III and ages 6–18 years, who were attending our service for clinically indicated 3-D gait analysis. Exclusion criteria were significant illnesses (such as major cardiac or respiratory disorders), injury or surgery within the last 6 months that may impact usual activity levels in the community or planned treatment following 3-DGA that precluded wearing of the monitor for a week.

The children were recruited when they attended a hospital clinic for their 3-DGA assessment. Written consent was obtained from each child’s parent or guardian and assent from the child.

A Step Watch™ Activity Monitor (Orthocare Innovations, Mountlake Terrace, WA, USA) was fitted to the less impaired lower limb using the strap according to manufacturer’s instructions. The monitor was then calibrated in clinic to each participant’s walking pattern. An accuracy check was performed by asking the child to walk at varying speeds in the clinic and manually correlating the triggered flashes from the internal LED light to the steps taken. Monitor calibration accuracy was established to manual counting and was achieved when greater than 95 % for all participants. All participants were given verbal and written instructions to wear the monitor for a continuous 7 day period, removing it only for sleeping, swimming, bathing and showering. Data from the monitors were downloaded after being returned to the principal investigator by mail. Data collection occurred during the year, with exclusion of school holidays.

### Data analysis

Previous work using the StepWatch™ Activity Monitor has found that both typically developing children and children with CP have lower and more varied activity levels on weekend days, possibly as the result of a less structured environment [22, 29]. We thus chose to analyse data only for the 5 week-days collected during the seven consecutive days of monitoring. The StepWatch™ Activity Monitor captures step activity of a single leg, so the step counts were doubled to obtain the overall step count.

In the first part of the data analysis, we applied increasingly stringent definitions of a valid day to the patient datasets and determined the number of participant datasets consequently excluded from the final analysis. These criteria were based on either a required minimum number of recorded steps in a 24 h monitoring period (starting at >100 steps and then 1000 steps, increased in 1000 step increments) or a minimum number of hours of recorded activity in a 24 h monitoring period (increased in intervals of 30 min).

In the second part of the data analysis, we assessed the performance variability of measures of total step count in the free living natural environment. To assess how different definitions of a ‘valid day’ affected performance variability, we used two definitions commonly found in the literature to determine inclusion or exclusion of data from analysis:

1. When the monitor had recorded at least 100 steps over a 24 h period of monitoring
2. When the monitor had recorded at least 10 h of activity with less than 2 h of no recorded activity over a 24 h period of monitoring

Bland and Altman analyses were used to quantitate the performance variability between day one and day two [30]. Intra-class correlation coefficients (ICC) were also calculated with IBM SPSS Statistics Version 21 (SPSS Inc., Chicago, IL) utilising the two-way random, absolute agreement model to determine the variability of measures of total step count between day one and day two, using the two above definitions for a valid day.

## Results

**Fig. 1:**
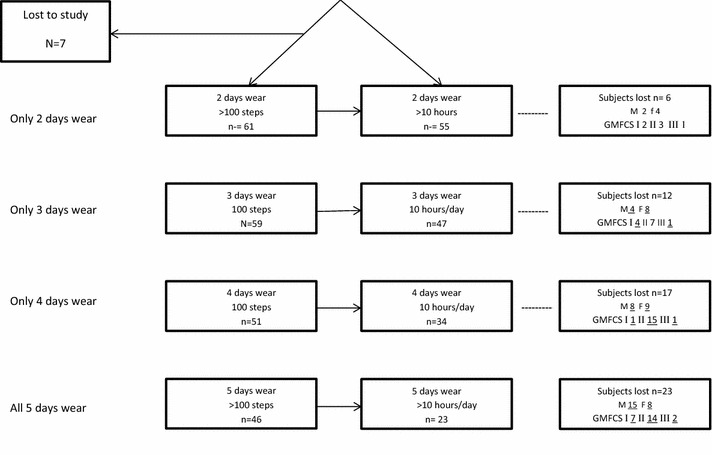
Flow diagram for 69 study participants

Table 1 presents the demographic data for all participants (n = 69) including gender, GMFCS level and sidedness of CP. The participant inclusions and exclusions that resulted from variations in (i) the definition of a ‘valid day’ and (ii) the number of valid days of consecutive monitoring required for final data analysis are shown in Fig. 1. Seven of the initially recruited 69 children had no recorded activity data and were excluded from the study. The reasons for the lack of recorded data were: no longer wanted to wear the monitor after enrolment in the study (n = 2), the monitor was lost (n = 4); and not wanting to repeat the assessment when no recorded activity was found on the returned monitor (presumed to have been worn upside down; n = 1). Of the remaining 62 participants with Step Watch data, one participant had only 1 day of recorded activity. For the remaining 61 participants, all had 2 or more days with >100 recorded steps in a 24 h period but only 55 participants had 2 or more valid days with ≥10 h in a 24 h period.

**Table 1:** Participant demographics

Number of participants	All participants recruited into study (n = 69)	All participants with recorded activity data (n = 62)	Valid day defined as >100 steps recorded activity over 24 h period (number of participants with valid days of monitoring)			Valid day defined as ≥10 h of recorded activity per 24 h period (number of participants with valid days of monitoring)		
			2 or more valid days (n = 61)	2 to 4 valid days (n = 15)	5 valid days (n = 46)	2 or more valid days (n = 55)	2 to 4 valid days (n = 32)	5 valid days (n = 23)
Age in years Median (range)	11 (6–18)	10 (6–16)	10 (6–16)	11 (6–16)	10 (6–16)	11 (6–16)	12 (6–16)	10 (7–13)
Male:female	33:36	31:31	31:30	7:8	24:22	29:26	17:5	12:11
GMFCS I;II;III	27; 37; 10	19; 35; 8	18; 35; 8	7; 7; 1	11; 28; 7	16; 32; 7	6; 24; 2	10; 8; 5
Bilateral:unilateral	38:31	36:26	36:25	7:8	29:17	32:23	20:12	12:11

*GMFCS* Gross Motor Function Classification System

**Fig. 2:**
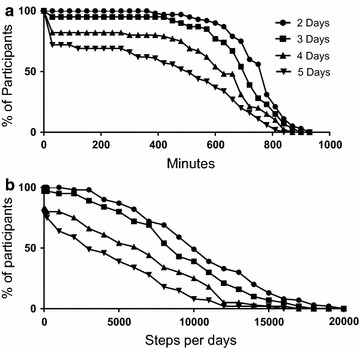
Variation in the percentage of participant datasets eligible for inclusion in final analysis. **a** By minimum wear time per 24 h period and the required number of days of monitoring. **b** By number of recorded steps per 24 h monitoring period and the required number of days of monitoring

Figure 2a, b show the percentage of participant datasets retained in the study analysis as the requirements for data inclusion are changed. If ≥600 min of recorded activity per 24 h period (with no less than 2 consecutive hours of no recorded activity) is defined as a valid day and the required number of valid days is only either 2 or 3 × 24 h periods, then 55 (90 %) and 47 (77 %) of the participant datasets are eligible for inclusion in the final analysis (Fig. 2a). However, if the same criterion for dataset inclusion is applied and the required number of valid days is extended up to 5 × 24 h periods, then only 23 (38 %) of the participant datasets meet the criteria for inclusion in the final analysis. If the required length of recorded activity is extended up to ≥720 min of recorded activity per 24 h period (with no less than 2 consecutive hours of no recorded activity), then the numbers of participants who achieved this wear time for 2 or 3 × 24 h periods over the week decreases to 37 (61 %) and 24 (39 %) respectively. Conversely if the required wear time is reduced to 480 min (8 h) per 24 h period and the total wear period is either 2 or 3 × 24 h periods, then 59 (97 %) and 55 (90 %) participant datasets will meet criteria for inclusion.

If the criterion for dataset inclusion in the final analysis was the number of recorded steps per 24 h period, then only a small number of participant datasets are excluded when the number of steps required per 24 h period is >100 steps. The number of participants who achieved this wear time for 2 or 3 × 24 h periods over the week are 61 (100 %) and 59 (97 %) respectively. Increasing the required wear period to 5 × 24 h periods, reduces the number of participant datasets meeting the criteria for inclusion in the final analysis to 46 (75 %). If the required number of recorded steps is increased to >1000 steps over a 24 h period, then the number of participants who achieved this wear time for 2 or 3 × 24 h periods over the week are not too dissimilar at 61 (100 %) and 57 (95 %) respectively. However, there was a progressive loss of participant datasets from the analysis as the number of steps per 24 h period increase above 3000 steps (Fig. 2b).

The demographics of the retained participants, including age, gender balance and sidedness of CP did not change significantly between 2 days and 5 valid days of monitoring for either ‘day’ definition, suggesting that changes in length of monitoring and ‘valid day’ definitions did not differentially affect retention of subgroups participants within the final analysis. However, the more stringent criteria for definition of a day of ≥10 h of recorded activity/24 h period led to a significant loss of children who functioned at GMFCS level II with a drop from 32 to 8 participants (75 % decrease), between 2 and 5 valid days of monitoring. This was significantly different from the retention of participants who functioned at GMFCS level II when the definition of a ‘valid day’ was >100 recorded steps over a 24 h period, with 28 of 35 participants being retained in the study (20 % decrease) (p < 0.012, Fisher’s exact test).

**Fig. 3:**
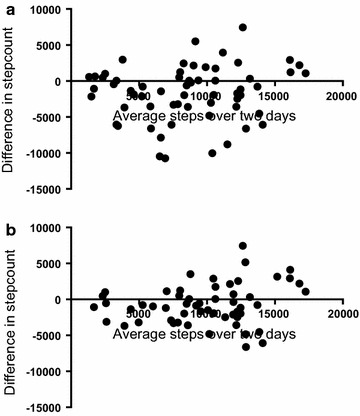
Bland–Altman plots. **a** For 2 days data with >100 steps per 24 h period. **b** For 2 days data >10 h per 24 h period

Overall, there was less variability in measurements of total step count between day one and day two when a valid day was described as ≥10 h recorded activity per 24 h period. Using this criterion for a valid day of monitoring, the ICC was 0.765 and the 95 % limits of agreement between the measures for 2 valid days were −6154 steps to 4797 steps, with a bias between day one and day two of −673 steps. Conversely, measurements of total step count between day one and day two were more variable when a valid day was described as >100 steps per 24 h period. Then the ICC was 0.62 and the 95 % limits of agreement between total step count for day one and day two were wider at −9055 steps to 5782 steps, with a bias between day one and day two of 1636 steps. These data are shown graphically in Fig. 3a, b. Table 2 shows the results of the Spearman Brown prophecy and the number of days of activity monitoring predicted to achieve ICCs of 0.7, 0.8 and 0.9 respectively.

**Table 2:** Performance variability of activity monitoring in the free living environment

	Number of children	Variability of 2 days of monitoring (ICC)	Predicted number of days of monitoring required to achieve an ICC of		
			0.7	0.8	0.9
>100 steps per 24 h period	61	0.620	2.8 days	4.9 days	11 days
≥10 h per 24 h period	55	0.765	1.4 days	2.5 days	5.5 days

*ICC* intra class correlation coefficient

## Discussion

The StepWatch™ Activity Monitor is widely used for assessing activity levels in a number of different populations, with total step count being the most frequently reported outcome. Monitoring is often for extended periods to capture different types of activity; however longer periods of monitoring also place increasing burden on the participants. In this study, using a convenience sample of children with CP, we found that many children do not achieve 5 full days of monitoring, regardless of how a valid day is defined. However, changes in the definition of a valid day made substantive differences to the number of participant datasets that were either retained or excluded from the final analysis. A low stringency criterion (>100 recorded steps/24 h period) led to retention of more participant datasets across all lengths of monitoring but had lower ICCs and more variability in step count between day one and day two. When a valid ‘day’ was defined as ≥10 h recorded step activity within a 24 h monitoring period, 2 complete days of monitoring led to an ICC of 0.765 and less variability in total step count measures. However, extending the numbers of valid days required to five consecutive days of ≥10 h activity monitoring per 24 h period, led to a 50 % drop in the numbers of participants with valid data-sets, with a disproportionate number of children functioning at GMFCS II level excluded from the analysis.

Our finding that many participants did not achieve 5 valid days of monitor recorded activity is not restricted to children with disabilities. In a large field-based, longitudinal study, Mattocks et al. required 7159 children aged 11 years wear an Actigraph monitor for seven days; however, 36 % wore the monitor for a valid 7 days and 56 % wore it for between 3 and 6 valid days [31]. Whether incomplete accelerometer data is included in, or excluded from, the final analysis has some potential to increase bias, as it likely reflects differences in how sub-groups of participants comply with study requirements. For example, Toftager et al. found that as the non-wear time of the monitor became shorter, more overweight and older adolescents were excluded [32]. In our study, children with CP, GMFCS level II were significantly more likely to be excluded as the criteria for study inclusion became more stringent. Children with CP GMFCS level II walk without walking aides, although have difficulty with stairs and activities on uneven ground. We are not certain why this group of children were differentially excluded but it is well known that children with CP can have associated behavioural and cognitive impairments as well as other medical conditions. These would potentially impact on study compliance [33]. This participant information was not available to this study but is worthy of further investigation.

We found in this study that monitoring for 2 consecutive days at ≥10 h per 24 h period, produced ICCs of 0.765, with the Spearman Brown prophecy predicting that 2.5 days of monitoring would be required to achieve an ICC of 0.8. A lower stringency criterion for dataset inclusion led to lower ICCs of 0.62. This finding is consistent with work by Rich et al. 2013 [34], who suggested that data from children with 2 days lasting >10 h of accelerometer monitoring is sufficient for monitoring in the typically developing population, achieving a reliability co-efficient of >0.8 calculated from the Spearman–Brown formula. In their study, shorter periods of monitoring per day (<10 h) necessitated more days of monitoring to achieve the same ICC. Addition of a weekend day did not alter the ICCs and was deemed not necessary.

Despite the ICC of 0.765 for the more stringent definition of a valid day, Bland–Altman analysis identified significant performance variability over 2 days with 95 % of repeated observations of total step counts expected to be within −6154 to 4797 steps of the first measure. The mean daily step count for those 55 participants was only 9870 steps, meaning that a very substantive change in daily total step count would be needed to prove efficacy of an intervention in a randomised trial. In practical terms, positive changes of less than this amount would be blurred by the background variability. It is thus unlikely that total step count would be a useful measure in a small study due to its variability, influenced by both personal and environmental factors.

Bland–Altman analyses do not determine the cause of variation or determine which measure is more accurate. The variability detected in total step count on a daily basis is likely a consequence of variation within participant activity levels over the school week coupled with missing data due to monitor misplacement or monitor removal and some degree of variation in sensitivity of step detection within the measurement device. A large study of 209 children with CP by Ishikawa et al. suggested that between a third and a half of the variance in total step count recorded by the StepWatch™ Activity Monitor was related to the participant’s functional ability and another third to half due to unquantifiable factors [28]. Only a small percentage of the variation was attributable to the day of measurement. This raises the question as to whether an ICC of 0.8 should be the goal and whether it is better to include all days with recorded activity and accept the wide variation from day or day or include only those datasets with longer periods of activity monitoring. The decision on this will likely vary, depending on the goal of the study but needs to be explicit in the study design. Certainly our data suggests that, as the stringency of the criterion for dataset inclusion increases, the more difficult it becomes to achieve complete datasets for all participants.

There were several limitations to this study. First there were only a small number of participants in the study, which may limit the generalizability of the results. Second, activity levels could have been underreported for several reasons, e.g. the monitor could not be worn when swimming and therefore may underreport physical activity as swimming has been found to be a frequent leisure physical activity for adolescents with CP [3]. Further, school-aged children in our country often remove footwear within the classroom so it is possible that the monitor was removed at intervals during the day, leading to under-counting of physical activity.

## Conclusions

In conclusion, how a ‘valid day’ is defined has significant impact on the size of the sample and which individual datasets are retained within the study analysis. Researchers need to balance the variability of the data collected by the StepWatch™ Activity Monitor against the burden to participants and the need to retain sufficient participants within a research study to achieve an adequate sample size. The variability in total step count from day to day is also significant in this group of children and makes it difficult to use the StepWatch™ Activity Monitor as a primary outcome measure in a small intervention trial. Researchers need to consider this variability to ensure the design of appropriately powered research studies.

## Abbreviations

CP: cerebral palsy
GMFCS: Gross Motor Function Classification System
CV: coefficient of variation
ICC: intra-class correlation coefficients

## References

[1] Oskoui M, Coutinho F, Dykeman J, Jette N, Pringsheim T (2013). An update on the prevalence of cerebral palsy: a systematic review and meta-analysis. Dev Med Child Neurol..

[2] Bjornson KF, Belza B, Kartin D, Logsdon R, McLaughlin JF (2007). Ambulatory physical activity performance in youth with cerebral palsy and youth who are developing typically. Phys Ther.

[3] Maher CA, Williams MT, Olds T, Lane AE (2007). Physical and sedentary activity in adolescents with cerebral palsy. Dev Med Child Neurol.

[4] Capio CM, Sit CH, Abernethy B, Rotor ER (2010). Physical activity measurement instruments for children with cerebral palsy: a systematic review. Dev Med Child Neurol.

[5] Palisano R, Rosenbaum P, Walter S, Russell D, Wood E, Galuppi B (1997). Development and reliability of a system to classify gross motor function in children with cerebral palsy. Dev Med Child Neurol.

[6] Palisano RJ, Rosenbaum P, Bartlett D, Livingston MH (2008). Content validity of the expanded and revised Gross Motor Function Classification System. Dev Med Child Neurol.

[7] Bodkin AW, Robinson C, Perales FP (2003). Reliability and validity of the Gross Motor Function Classification System for cerebral palsy. Pediatr Phys Ther..

[8] Verschuren O, Darrah J, Novak I, Ketelaar M, Wiart L (2014). Health-enhancing physical activity in children with cerebral palsy: more of the same is not enough. Phys Ther.

[9] Priego Quesada JI, Lucas-Cuevas AG, Llana-Belloch S, Perez-Soriano P (2014). Effects of exercise in people with cerebral palsy. A review. J Phys Educ Sport.

[10] Bjornson KF, Yung D, Jacques K, Burr RL, Christakis D (2012). StepWatch stride counting: accuracy, precision, and prediction of energy expenditure in children. J Pediatr Rehabil Med..

[11] Riner WF, Sellhorst SH (2013). Physical activity and exercise in children with chronic health conditions. J Sport Health Sci.

[12] Bjornson KF (2005). Physical activity monitoring in children and youths. Pediatr Phys Ther..

[13] Christy JB, Chapman CG, Murphy P (2012). The effect of intense physical therapy for children with cerebral palsy. J Pediatr Rehabil Med..

[14] Wilson NC, Mackey AH, Naude Y, Donavon J, Stott NS. Pilot of study of the short term impact of lower limb orthopaedic surgery on children with cerebral palsy across the International Classification of Functioning Disability and Health. 2015. **(Manuscript prepared for publication)**.

[15] Busse ME, van Deursen RW, Wiles CM (2009). Real-life step and activity measurement: reliability and validity. J Med Eng Technol.

[16] Sandroff BM, Motl RW, Pilutti LA, Learmonth YC, Ensari I, Dlugonski D (2014). Accuracy of StepWatch and ActiGraph accelerometers for measuring steps taken among persons with multiple sclerosis. PLoS ONE.

[17] Fulk GD, Combs SA, Danks KA, Nirider CD, Raja B, Reisman DS (2014). Accuracy of 2 activity monitors in detecting steps in people with stroke and traumatic brain injury. Phys Ther.

[18] Jenkins S, Hill K, Cindy Ng LW (2012). Accuracy and responsiveness of the stepwatch activity monitor and ActivPAL in patients with COPD when walking with and without a rollator. Disabil Rehabil.

[19] Silcott NA, Bassett DR, Thompson DL, Fitzhugh EC, Steeves JA (2011). Evaluation of the Omron HJ-720ITC pedometer under free-living conditions. Med Sci Sports Exerc.

[20] Mitchell LE, Ziviani J, Boyd RN (2015). Variability in measuring physical activity in children with cerebral palsy. Med Sci Sports Exerc.

[21] Corder K, Ekelund U, Steele RM, Wareham NJ, Brage S (2008). Assessment of physical activity in youth. J Appl Physiol.

[22] Song KM, Bjornson KF, Cappello T, Coleman K (2006). Use of the StepWatch activity monitor for characterization of normal activity levels of children. J Pediatr Orthop.

[23] Tudor-Locke C, Johnson WD, Katzmarzyk PT (2009). Accelerometer-determined steps per day in US adults. Med Sci Sports Exerc.

[24] Balemans AC, van Wely L, Middelweerd A, van den Noort J, Becher JG, Dallmeijer AJ (2014). Daily stride rate activity and heart rate response in children with cerebral palsy. J Rehabil Med.

[25] Van Wely L, Dallmeijer AJ, Balemans AC, Zhou C, Becher JG, Bjornson KF (2014). Walking activity of children with cerebral palsy and children developing typically: a comparison between the Netherlands and the United States. Disabil Rehabil.

[26] Herrmann SD, Barreira TV, Kang M, Ainsworth BE (2014). Impact of accelerometer wear time on physical activity data: a NHANES semisimulation data approach. Br J Sports Med.

[27] Bassett DR, Wyatt HR, Thompson H, Peters JC, Hill JO (2010). Pedometer-measured physical activity and health behaviors in U.S. adults. Med Sci Sports Exerc.

[28] Ishikawa S, Kang M, Bjornson KF, Song K. Reliably measuring ambulatory activity levels of children and adolescents with cerebral palsy. Arch Phys Med Rehabil. 2012;94(1):132–7. 10.1016/j.apmr.2012.07.027 PMC3645002 22892322

[29] van Wely L, Becher JG, Balemans AC, Dallmeijer AJ (2012). Ambulatory activity of children with cerebral palsy: which characteristics are important?. Dev Med Child Neurol.

[30] Bland JM, Altman DG (1996). Measurement error. BMJ..

[31] Mattocks C, Ness A, Leary S, Tilling K, Blair SN, Shield J (2008). Use of accelerometers in a large field-based study of children: protocols, design issues, and effects on precision. J Phys Act Health..

[32] Toftager M, Kristensen PL, Oliver M, Duncan S, Christiansen LB, Boyle E (2013). Accelerometer data reduction in adolescents: effects on sample retention and bias. Int J Behav Nutr Phys Act..

[33] Novak I, Hines M, Goldsmith S, Barclay R (2012). Clinical prognostic messages from a systematic review on cerebral palsy. Pediatrics.

[34] Rich C, Geraci M, Griffiths L, Sera F, Dezateux C, Cortina-Borja M (2013). Quality control methods in accelerometer data processing: defining minimum wear time. PLoS ONE.

